# COGNITIVE TRAINING FOR OLDER CHINESE IMMIGRANTS IN THE US: AN EXPERIENCE-BASED CO-DESIGN APPROACH

**DOI:** 10.1093/geroni/igac059.068

**Published:** 2022-12-20

**Authors:** Hanzhang Xu, Eleanor McConnell, John Myers, Bei Wu

**Affiliations:** Duke University, Durham, North Carolina, United States; Duke University, Durham, North Carolina, United States; Duke University School of Nursing, Durham, North Carolina, United States; New York University, New York, New York, United States

## Abstract

Older Chinese Americans face many socioeconomic barriers including limited English proficiency, low educational levels, and limited access to care. These socioeconomic disadvantages not only contribute to an increased risk of developing dementia but also worsen inequitable access to effective strategies to promote cognitive health. Cognitive training is shown to be beneficial to maintain or enhance cognitive function. However, most prior interventions were tested exclusively in non-Hispanic Whites. To address this gap, we aim to adapt empirically supported cognitive training activities into a culturally and linguistically relevant mHealth cognitive training intervention. The adaptation process of the cognitive training includes focus groups (n=6/group) with older Chinese Americans (3 groups) and adult children (2 groups) to adapt cognitive training components to our target population. We will then organize an experience-based co-design workshop to further refine the intervention. Engaging end-users early will optimize the development of a culturally and linguistically relevant cognitive training intervention.

